# Structure and Properties of Bioactive Titanium Dioxide Surface Layers Produced on NiTi Shape Memory Alloy in Low-Temperature Plasma

**DOI:** 10.3390/mi15070886

**Published:** 2024-07-06

**Authors:** Justyna Witkowska, Tomasz Borowski, Krzysztof Kulikowski, Karol Wunsch, Jerzy Morgiel, Jerzy Sobiecki, Tadeusz Wierzchoń

**Affiliations:** 1 Faculty of Materials Science and Engineering, Warsaw University of Technology, 02-507 Warsaw, Poland; tomasz.borowski@pw.edu.pl (T.B.); krzysztof.kulikowski@pw.edu.pl (K.K.); karol.wunsch.dokt@pw.edu.pl (K.W.); jerzy.sobiecki@pw.edu.pl (J.S.); tadeusz.wierzchon@pw.edu.pl (T.W.); 2 Institute of Metallurgy and Materials Science, Polish Academy of Sciences, 30-059 Krakow, Poland; j.morgiel@imim.pl

**Keywords:** NiTi shape memory alloys, glow-discharge oxidizing, structure, biocompatibility, bioactivity

## Abstract

Background: The NiTi alloy, known for its shape memory and superelasticity, is increasingly used in medicine. However, its high nickel content requires enhanced biocompatibility for long-term implants. Low-temperature plasma treatments under glow-discharge conditions can improve surface properties without compromising mechanical integrity. Methods: This study explores the surface modification of a NiTi alloy by oxidizing it in low-temperature plasma. We examine the impact of process temperatures and sample preparation (mechanical grinding and polishing) on the structure of the produced titanium oxide layers. Surface properties, including topography, morphology, chemical composition, and bioactivity, were analyzed using TEM, SEM, EDS, and an optical profilometer. Bioactivity was assessed through the deposition of calcium phosphate in simulated body fluid (SBF). Results: The low-temperature plasma oxidization produced titanium dioxide layers (29–55 nm thick) with a predominantly nanocrystalline rutile structure. Layer thickness increased with extended processing time and higher temperatures (up to 390 °C), though the relationship was not linear. Higher temperatures led to thicker layers with more precipitates and inhomogeneities. The oxidized layers showed increased bioactivity after 14 and 30 days in SBF. Conclusions: Low-temperature plasma oxidation produces bioactive titanium oxide layers on NiTi alloys, with a structure and properties that can be tuned through process parameters. This method could enhance the biocompatibility of NiTi alloys for medical implants.

## 1. Introduction

NiTi shape memory alloys (SMAs), a unique class of materials with shape memory and superelasticity properties, have garnered significant attention in the medical field due to their biocompatibility and corrosion resistance [1,2,3,4]. The inherent shape memory and superelasticity properties of NiTi SMAs allow them to undergo deformation and recover their original shape, making them suitable for implants subjected to mechanical stresses within the body [5], especially long-term bone implants. Materials intended for medical implants must demonstrate excellent biocompatibility, minimizing adverse reactions when in contact with biological tissues. This characteristic is crucial for the long-term success of medical implants [6]. The corrosion resistance of the material must ensure the durability of implants within the harsh physiological environment. This property is vital for maintaining the structural integrity of implants over their lifespan [7].

Shape memory alloys have also garnered significant interest in the realm of micromachines, offering a plethora of potential applications, notably in shape memory alloy actuators. These materials’ properties make them ideal candidates for various microscale actuation mechanisms. One promising application lies in the use of microactuators for biomedical devices. NiTi SMAs have been utilized in the development of microvalves and microgrippers for minimally invasive surgeries and targeted drug delivery systems [8]. The biocompatibility of NiTi further enhances its suitability for such applications, ensuring minimal adverse reactions within the human body. In the field of microfluidics, NiTi SMA actuators have shown promise in the creation of micropumps and microvalves for precise fluid control in lab-on-a-chip devices [9]. These actuators enable rapid and reversible changes in fluid flow, facilitating tasks such as mixing, sorting, and metering within microfluidic systems. Moreover, NiTi SMA-based microactuators find applications in adaptive optics and micro-opto-electro-mechanical systems (MOEMS). By integrating SMA-based deformable mirrors or lenses into optical systems, researchers have achieved dynamic control over focal length and aberrations, leading to advancements in adaptive optics for astronomy and high-resolution imaging [10]. Furthermore, NiTi SMAs hold promise in the field of micro-electro-mechanical systems (MEMS) for the precise positioning and manipulation of microscale components. These actuators can be employed in microgrippers to handle delicate objects in manufacturing processes or for microassembly tasks involving electronic and optical devices [11]. Therefore, the unique combination of mechanical properties, biocompatibility, and responsiveness to external stimuli positions NiTi SMAs as a versatile material for driving innovation in microscale actuation systems [12].

One of the primary challenges associated with NiTi SMA’s application in medicine is the potential release of nickel ions, which may lead to allergic reactions and cytotoxicity [13,14,15]. Researchers are actively investigating strategies to minimize nickel ion release [16]. Various surface engineering methods have been explored as surface modification techniques to enhance the biocompatibility of NiTi SMA implants and control the biological properties of the surface depending on the intended use [7,17]. Thin films, layers, or coatings can improve wear resistance and reduce ion release, addressing concerns related to the alloy’s nickel content [13,18]. An exemplary hydroxyapatite (HA) coating, known for its osteoconductive properties, is applied to NiTi SMA surfaces to promote bone formation and improve osseointegration. This surface modification strategy aims to address challenges related to surface roughness and enhance the bioactivity of the implants [19]. Diamond-like carbon (DLC) coatings, due to their hemocompatible properties, are recommended for use in implants in contact with blood [20]. Despite the advantages of the developed techniques, challenges such as the adhesion of coatings, the possibility of controlling the structure and properties through technological parameters of processes, and the possibility of using low temperatures that guarantee the preservation of the specific properties of the shape memory alloy [21] need to be addressed. Surface treatments in low-temperature glow-discharge plasma meet all the above-mentioned requirements and, in addition, allow for the production of thin, nanometric-thick diffusion layers whose properties can be shaped depending on the needs of the specific implant application, paving the way for improved medical outcomes.

Our previous research indicates very promising properties of oxidized [22], nitrided [23], or oxynitrided [24] layers produced in low-temperature plasma in the context of shaping the properties of the NiTi alloy for medical applications. However, their bioactivity, understood as the ability to undergo the biomimetic deposition of calcium phosphate while incubating in a simulated body fluid (SBF) solution, is not yet well-described [25]. This is an important and current cognitive aspect with very practical implications. Therefore, the aim of this work was to describe the effects of different process temperatures and the method of preparing the sample surface (mechanical grinding and polishing) on the structure of the produced oxidized surface layers and their properties, including surface topography, surface morphology, chemical composition, and bioactivity.

## 2. Materials and Methods

The researched material comprised a commercially available NiTi shape memory alloy with a chemical composition of 50.8 %at. Ni (Ti—rest). Disc-shaped samples, with a diameter of ø8 mm and a thickness of 1 mm, were cut from a bar and utilized in the tests. The samples underwent mechanical grinding with up to 1200 grit sandpaper or mechanical polishing in a diamond suspension with a grain size of 1 μm and silicon oxide with a grain size of 20 nm and were subsequently degreased in acetone.

**Figure 1:**
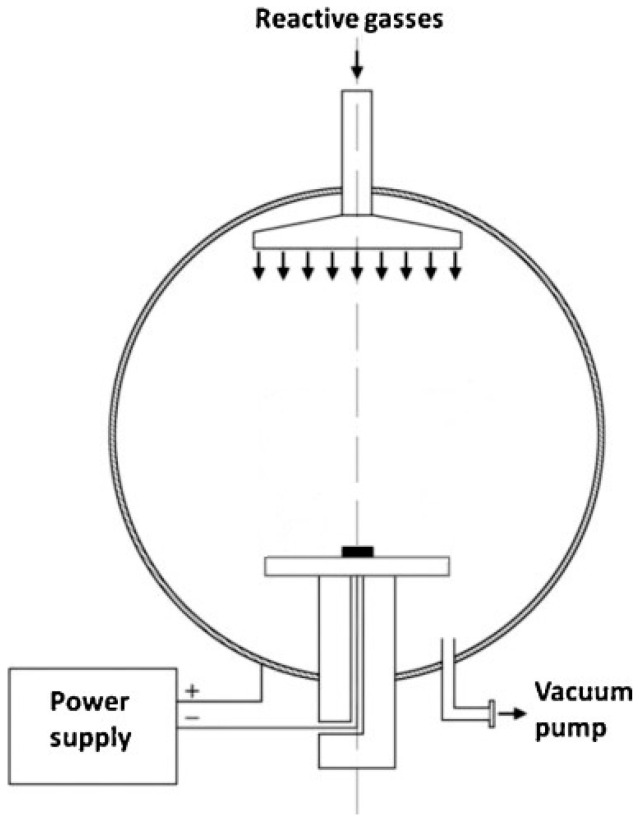
The schematic representation of the reaction chamber for glow-discharge treatments.

Glow-discharge oxidizing processes were conducted for 60 min in a pure oxygen DC plasma at a chamber operating pressure of 1.6 mbar and temperatures of 290 °C (ground and polished samples), 190 °C (ground samples), and 390 °C (ground samples). The schematic representation of the reaction chamber for glow-discharge treatments, constructed by members of the research team, is presented in Figure 1. The process at 290 °C served as a reference, having been predominantly used in earlier exploratory research. The processes at 190 °C and 390 °C were referred to as reduced and increased temperatures, respectively. The temperature was monitored using a pyrometer, and before oxidizing, specimens were heated to the process temperature in nitrogen plasma (N_2_ atmosphere with 99.999% purity).

Microstructure investigations of the produced surface layers were conducted using a TECNAI SuperTWIN (200 kV) FEG transmission electron microscope (TEM) (FEI, Eindhoven, The Netherlands) capable of working in scanning mode (STEM). Nanoscale local chemical analysis was performed using an integrated EDAX energy-dispersive microanalysis (EDS) system (EDAX, Tilburg, The Netherlands).

For surface topography examinations, a Wyko NT 9300 scanning optical profilometer (Veeco, Plainview, NY, USA) was employed. Roughness parameters were measured in 480 × 640 µm areas, with five different observation areas measured for each sample, and mean and standard deviations were calculated.

NiTi samples in the initial state and with oxidized surface layers were incubated in SFB solution, prepared according to Kokubo [26]. Incubation periods of 14 and 30 days at 37 °C were applied, with the solution changed every 5 days. A 14-day incubation was conducted for all sample types, while a 30-day incubation was carried out only for mechanically ground ones.

The surface morphology of the samples before and after incubation in the SBF solution was imaged using a scanning electron microscope (S-3500N, Hitachi, Tokio, Japan) with an acceleration voltage of 15 keV in SE (secondary electron) mode. An EDS attachment was utilized for the analysis of the chemical composition of the biomimetic apatite layers on the sample surface after incubation.

## 3. Results and Discussion

**Figure 2:**
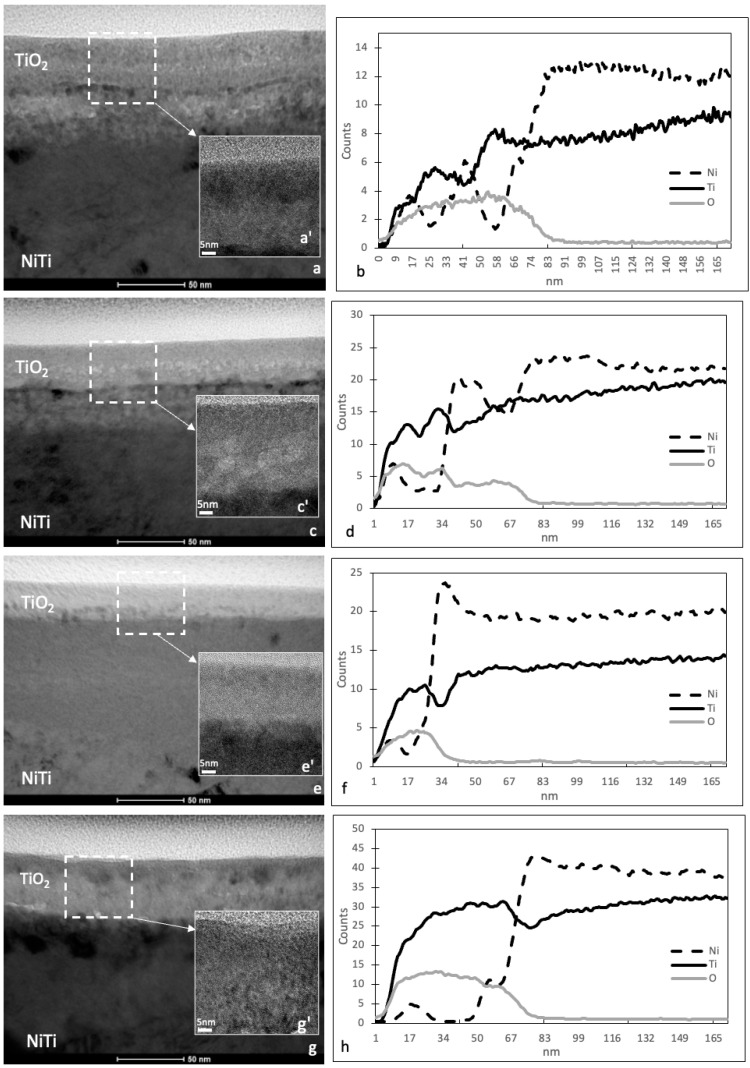
Microstructure of TiO_2_ surface layers produced on NiTi alloy: at 290 °C on ground surface (**a**), at 290 °C on polished surface (**c**), at 190 °C on ground surface (**e**), and at 390 °C on ground surface (**g**); corresponding HRSTEM images of TiO_2_ layers (**a’**,**c’**,**e’**,**g’**) and corresponding linear distributions of elements in the TiO_2_ layers (**b**,**d**,**f**,**h**).

Oxidizing processes were utilized to create surface layers on the NiTi alloy, displaying varying thickness and structures depending on the process temperature and surface preparation. Transmission electron microscope (TEM) images depict the cross-sections of the oxidized surface layers formed in low-temperature plasma on the NiTi shape memory alloy (Figure 2a,c,e,g). Additionally, high-resolution scanning transmission electron microscopy (HRSTEM) images were taken from selected regions of the oxidized layers (Figure 2a’,c’,e’,g’). Consistent with our previous investigations [24], layers produced at 290 °C primarily comprise titanium dioxide (TiO_2_) rutile-type with a nanocrystalline structure. However, HRSTEM analyses suggest the presence of amorphous oxide phases, particularly in layers produced at lower temperatures (Figure 2e,e’). The approximate thickness of a titanium oxide layer is 52 nm for the layer produced at 290 °C on a ground surface, 34 nm for the layer produced at 290 °C on a polished surface, 29 nm for the layer produced at 190 °C, and 55 nm for the layer produced at 390 °C. In each case, a transition zone with different thicknesses can be observed under the titanium dioxide layers: approximately 12–18 nm for the layer produced at 290 °C on a ground surface, approximately 25 nm for the layer produced at 290 °C on a polished surface, approximately 6–10 nm for the layer produced at 190 °C, and approximately 25 nm for the layer produced at 390 °C. The elemental composition of the layer is further supported by the linear distribution of elements obtained from energy-dispersive X-ray spectroscopy (EDS) (Figure 2b,d,f,h). This confirms the presence of titanium dioxide in the oxidized layer but also indicates small amounts of nickel oxides in the surface layer and larger amounts of nickel oxides in the transition zones under the oxidized surface layers.

**Figure 3:**
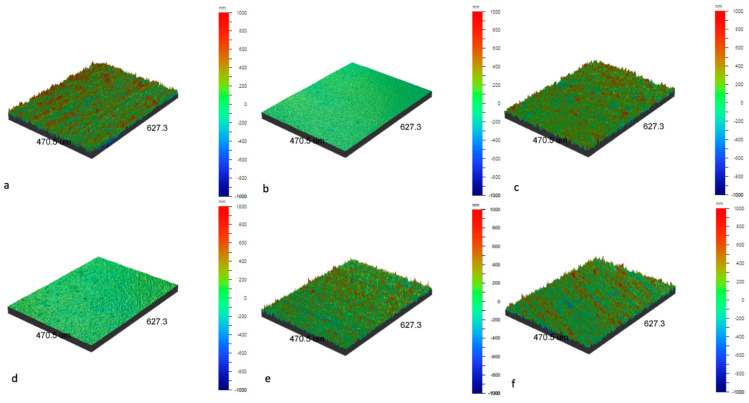
Surface topography of NiTi alloy in initial state on a ground surface (**a**), polished surface (**b**), and with TiO_2_ surface layers produced at 290 °C on ground surface (**c**), at 290 °C on polished surface (**d**), at 190 °C on ground surface (**e**), and at 390 °C on ground surface (**f**), as visualized by optical profilometer.

Low-temperature plasma treatments have influenced the surface topography of the NiTi alloy. An examination of roughness parameters (Table 1) and optical profilometer images (Figure 3) reveals that, for mechanically ground samples, oxidation processes resulted in enhanced surface smoothness. The most significant difference in roughness parameters was observed for samples oxidized at a reduced temperature, with a lesser effect for samples oxidized at 290 °C and 390 °C. In the case of mechanically polished samples, which are initially very smooth, the oxidation process in low-temperature plasma led to a subtle development of their surface. Nevertheless, it remained significantly smoother than that of mechanically polished samples, even after undergoing oxidation processes. It can be concluded that the topography of the samples is primarily influenced by the surface preparation before the process. The samples, post-glow-discharge processes, still exhibit their original surface topography, retaining minor changes characteristic of phenomena occurring during oxidation, such as the formation of titanium oxide layers, particularly changes characteristic of areas with increased energy, such as scratches created after the grinding process.

The success of bone implants relies on establishing a favorable interface between the implant material and the surrounding biological environment. Recent advancements in biomaterial research underscore the crucial role of surface topography in modulating cellular behavior and promoting optimal tissue integration. Surface topography significantly influences cell adhesion and proliferation. Micro- and nanoscale features on implant surfaces have been shown to enhance initial cell attachment and promote subsequent proliferation, contributing to improved tissue integration [27]. Achieving an optimal surface finish is essential to promote osseointegration and reduce the risk of complications [28]. Certain surface topographies, such as grooves, pits, and hierarchical structures, have been reported to induce the osteogenic differentiation of mesenchymal stem cells, leading to enhanced bone formation around implants [29]. Surface topography plays a critical role in the process of osseointegration, influencing the degree of bone–implant contact and overall implant stability. Micro- and nanostructured surfaces have been demonstrated to promote faster and stronger osseointegration compared to smooth surfaces [30]. Incorporating bioactive coatings or modifying implant surfaces with nanotopographical features has shown promise in enhancing bone–implant interactions. These strategies aim to mimic the natural extracellular matrix and create a microenvironment conducive to cell adhesion and differentiation [31]. Despite significant progress, challenges remain in fully understanding the complex interactions between surface topography and cellular responses. Further research is needed to explore the long-term effects of specific topographical features and their impact on implant performance in vivo.

**Figure 4:**
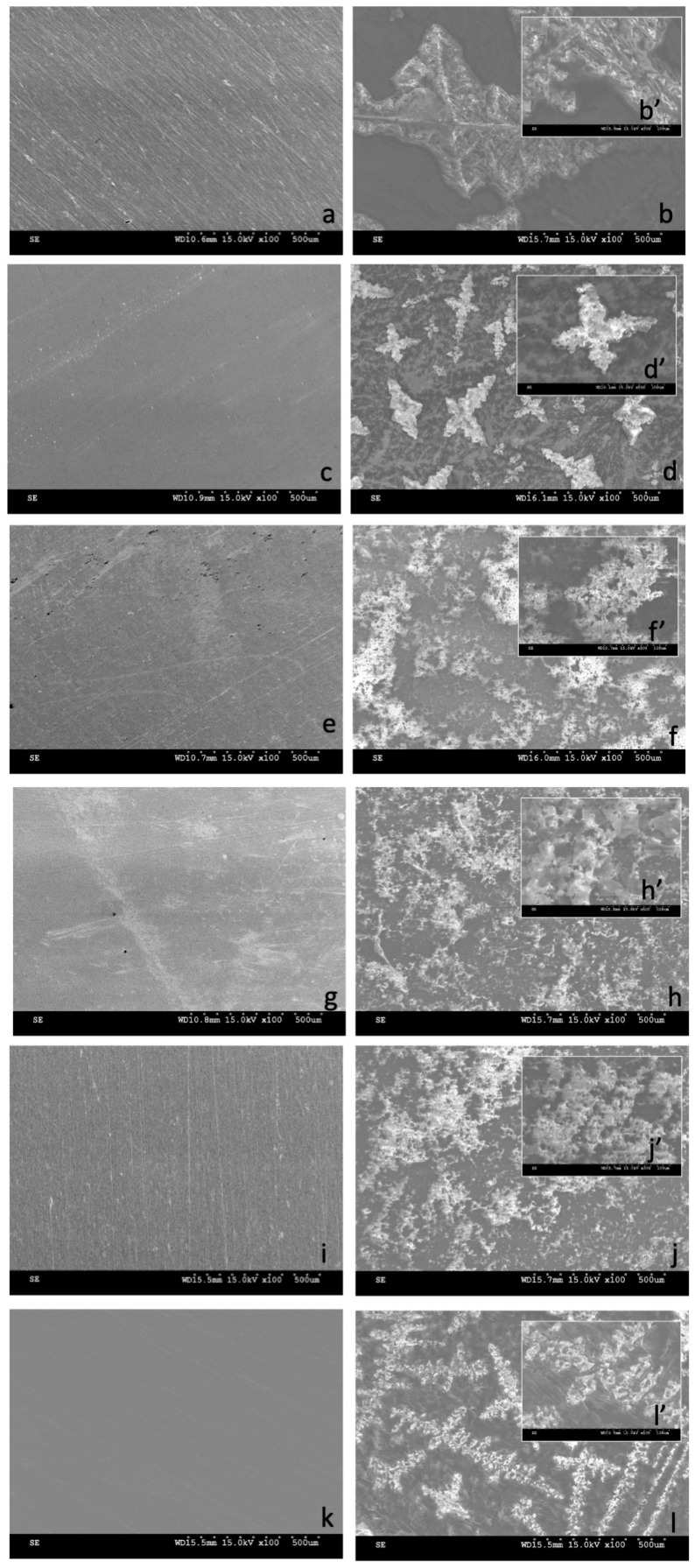
Surface morphology of NiTi alloy in the initial state: ground surface (**a**,**b**,**b’**), polished surface (**c**,**d**,**d’**), and with TiO_2_ surface layers produced at 290 °C on ground surface (**e**,**f**,**f’**), at 290 °C on polished surface (**g**,**h**,**h’**), at 190 °C on ground surface (**i**,**j**,**j’**), and at 390 °C on ground surface (**k**,**l**,**l’**), both before (**a**,**c**,**e**,**g**,**i**,**k**) and after (**b**,**b’**,**d**,**d’**,**f**,**f’**,**h**,**h’**,**j**,**j’**,**l**,**l’**) 14-day incubation in SBF solution: SEM images magnified ×100 (**a**–**l**), ×500 (**b’**,**d’**,**f’**,**h’**,**j’**,**l’**).

The surface morphology of the NiTi alloy in its initial state and after surface treatments was visualized through scanning electron microscope (SEM) observations (Figure 4), both before and after a 14-day incubation in simulated body fluid (SBF) solution. The surface morphology before sample incubation aligns with surface topography measurements; samples after oxidation processes reflect the topography of the substrate, which is primarily determined by the method of surface preparation—either grinding or polishing—with slight modifications from oxidation processes in low-temperature plasma.

After 14 days of incubation, deposits were observed on samples without a layer, yet these were identified as NaCl crystals rather than calcium phosphate deposits, as confirmed by EDS tests. This occurrence was observed for both ground and polished samples, suggesting the limited bioactivity of samples without an oxidized layer. In contrast, samples with oxidized layers after 14 days displayed numerous apatite deposits, consisting of calcium, phosphorus, sodium, magnesium, potassium, and oxygen, among others. Notably, the only type of surface layer not showing the formation of calcium phosphate deposits after 14 days was the oxidized layer formed at an elevated temperature (390 °C). Deposits that were similar, in terms of morphology and chemical composition, to those observed on samples in the initial state were noted for this layer.

A chemical composition analysis of the observed apatite deposits (Table 2) confirmed significant amounts of calcium, phosphorus, and oxygen for samples with oxidized layers formed at temperatures of 290 °C (both on ground and polished surfaces) and 190 °C. The sample with a layer produced at a temperature of 190 °C exhibited the highest content of both calcium and phosphorus, while the highest Ca/P ratio was found for the layer produced at a temperature of 290 °C on the ground surface, a slightly lower ratio was found on the polished surface, and the lowest ratio was observed for the one produced at a reduced temperature. Samples without layers and those with layers formed at elevated temperatures contained negligible amounts of Ca and P.

**Figure 5:**
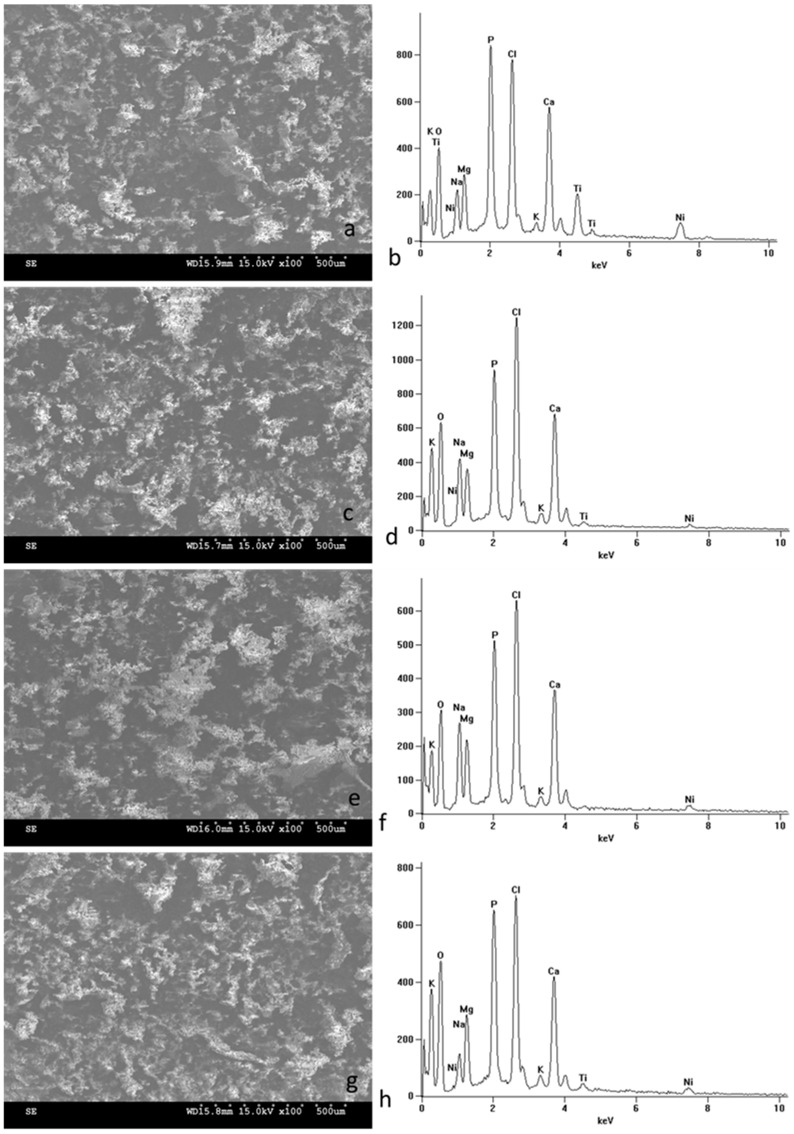
Surface morphology of NiTi alloy in the initial state (**a**) and with TiO_2_ surface layers produced at 290 °C (**c**), at 190 °C (**e**), and at 390 °C (**g**) after 30-day incubation in SBF solution: SEM images magnified ×100. Corresponding chemical compositions (**b**,**d**,**f**,**h**) obtained by EDS from the entire image area after 14-day incubation in SBF solution.

For further research, the incubation in SBF solution was extended to 30 days, with only ground samples selected for comparison. SEM microscope imaging of the surfaces of the samples after prolonged incubation (Figure 5) revealed a uniform layer of apatite deposits covering the entire surface for all observed samples. EDS spectra and chemical composition analyses from the entire observed surface and specific points confirmed the high contents of calcium, phosphorus, and oxide in the formed biomimetic deposits (Table 3). The Ca/P ratio ranged from 0.98 to 1.15 for whole-surface analyses and from 0.95 to 1.76 for spot analyses.

The types of calcium phosphate that can be generated in biomimetic processes include β-tricalcium phosphate (β-TCP), hydroxyapatite (usually calcium-deficient hydroxyapatite), dicalcium phosphate dihydrate/Brushite, amorphous calcium phosphate (ACP), dehydrated dicalcium phosphate (DCPC) [6,32,33,34,35], etc. Typically, the constituent elements of diverse calcium phosphate compositions are blended and firmly interlocked with each other. Although the morphology of the formed apatite deposits is similar, differences exist in the Ca/P ratio for individual types of layers. Therefore, it can be inferred that their phase composition is distinct, with each layer being characterized by its unique mixture of the several types of calcium phosphates that are formed. Further research into the precise phase composition of these biomimetic coatings is advisable and will be continued at a later stage of the research.

At the same time, it is possible to observe a faster formation of apatite deposits on surfaces with oxidized layers produced at temperatures below 300 °C compared to the initial state of the NiTi alloy. These observations align with the literature, which indicates the good bioactivity of titanium oxide layers [36,37,38]. Within simulated biological conditions at pH 7.4, the TiO_2_ surface exhibits predominantly negative characteristics, creating an electrostatic environment conducive to attracting oppositely charged ions, such as calcium. Subsequently, the positively charged Ca^2+^ undergoes a reaction with negatively charged PO_4_^3−^ and CO_3_^2−^, leading to the formation of a surface layer containing Ca–P. Over time, this layer may crystallize into a bone-like apatite [39]. The similarity of the coating formation environment to that of natural apatite suggests that coatings produced through this method may offer superior bone-bonding capabilities compared to conventional techniques like plasma spraying [39]. An inherent advantage of employing a biomimetic approach for coating metal implants with bone-like apatite layers lies in its emulation of the natural process by which hydroxyapatite bone crystals develop in the body. Consequently, the generated coatings consist of smaller crystal units, which are more readily susceptible to degradation by osteoclasts compared to the larger ceramic particles produced through different techniques [40]. Biomimetic processes are, therefore, a good reflection of the phenomena occurring in the body after the implantation of the biomaterial [26]. The lower bioactivity of layers produced at elevated temperatures may be surprising, but may be related to the influence of precipitates and inhomogeneities visible in cross-sections in bright-field TEM images, which influence the interaction with the ions of the solution. After 30 days of incubation, this layer is also covered with a coating of biomimetic calcium phosphates.

## 4. Conclusions

To summarize, the current study demonstrates the following:

- Variations in the temperature of the oxidation process in low-temperature glow discharge plasma, while maintaining consistent process duration, significantly impact the thickness and structure of the resulting titanium oxide layers.
- The nature of the titanium oxide layer and the nickel-enriched transition zone beneath the oxidized layer is also influenced by the surface preparation, such as mechanical polishing or mechanical grinding.
- Oxidized layers exhibit increased bioactivity compared to the alloy in its initial state.
- The distinct structure of these layers results in a varied composition of biomimetic deposits composed of calcium phosphates.
- The surface preparation before the process did not significantly affect the level of bioactivity in the layers but did influence the phase composition of calcium phosphates. The precise identification of compounds in the future will enable the accurate association of a specific phase composition with a given type of layer.
- All tested materials, including those in their initial state, display a high level of bioactivity in the long term (30 days).
- The expedited formation of biomimetic calcium phosphates may contribute to the potentially enhanced osseointegration of the material when used for bone implants. Increased bioactivity is thus promising in the context of broadening the application of NiTi alloys in medicine. Consequently, oxidation processes in low-temperature plasma can be regarded as a crucial aspect of material solutions for constructing biocompatible NiTi alloy implants. As this study has a preliminary character, further investigations are planned to comprehensively assess the long-term effectiveness of our surface treatment.

## Figures and Tables

**Table 1:** Surface roughness parameters for NiTi alloys in the initial state for ground and polished surfaces and with TiO_2_ surface layers: at 290 °C on ground surface, at 290 °C on polished surface, at 190 °C on ground surface, and at 390 °C on ground surface, as measured by optical profilometer.

	NiTi (Ground Surface)	NiTi (Polished Surface)	NiTi + TiO_2_ (290 °C, Ground Surface)	NiTi + TiO_2_ (290 °C, Polished Surface)	NiTi + TiO_2_ (190 °C, Ground Surface)	NiTi + TiO_2_ (390 °C, Ground Surface)
Ra [nm]	197.1 ± 6.6	20.5 ± 4.3	170.0 ± 6.3	30.1 ± 1.9	145.4 ± 2.3	172.9 ± 5.8
Rq [nm]	254.8 ± 9.9	25.9 ± 5.1	221.9 ± 6.7	39.7 ± 2.3	192.9 ± 2.5	223.3 ± 7.8
Rz [nm]	2567.1 ± 294.5	336.2 ± 58.4	2301.6 ± 67.3	690.4 ± 53.6	2222.0 ± 54.3	2596.7 ± 137.5
Rt [nm]	3279.4 ± 664.5	580.6 ± 180.0	2829.5 ± 224.6	954.5 ± 140.4	2650.1 ± 248.5	3647.5 ± 478.9

Ra—arithmetic average of absolute values of profile heights over evaluated length; Rq—root mean square average of the profile heights over evaluated length; Rz—the absolute vertical distance between the maximum profile peak height and the maximum profile valley depth along the sampling length; Rt—total height of the roughness profile: difference between height of the highest peak and depth of the deepest valley along the sampling length

**Table 2:** The amount of calcium, phosphorus, and oxygen expressed in atomic %, determined by EDS measurements for non-oxidized NiTi and NiTi with a TiO_2_ surface layers after 14 days of SBF incubation. Results obtained for measurements from the image area and the average of three point measurements.

14 Days in SBF	Ca (%at.)		P (%at.)		O (%at.)		Ca/P	
Place of Analysis	Image Area	Point	Image Area	Point	Image Area	Point	Image Area	Point
NiTi (mechanically ground surface)	0.14	0.44 ± 0.05	0.33	0.14 ± 0.02	20.79	2.42 ± 0.09	0.42	3.14
NiTi (mechanically polished surface)	-	-	0.32	1.57 ± 0.09	15.96	29.94 ± 0.56	-	-
NiTi + TiO_2_ (290 °C, mechanically ground surface)	16.67	17.06 ± 0.49	12.48	11.67 ± 0.35	36.68	19.08 ± 1.29	1.34	1.46
NiTi + TiO_2_ (290 °C, mechanically polished surface)	18.25	27.48 ± 0.15	13.73	21.10 ± 0.12	32.93	35.81 ± 0.67	1.33	1.30
NiTi + TiO_2_ (190 °C, mechanically ground surface)	20.22	27.39 ± 0.18	24.70	22.36 ± 0.14	37.30	21.28 ± 0.83	0.81	1.23
NiTi + TiO_2_ (390 °C, mechanically ground surface)	0.13	0.54 ± 0.02	0.34	0.02 ± 0.02	21.81	0.70 ± 0.07	0.10	-

**Table 3:** The amount of calcium, phosphorus, and oxygen expressed in atomic %, determined by EDS measurements for non-oxidized NiTi and NiTi with a TiO_2_ surface layers after 30 days of SBF incubation. Results obtained for measurements from the image area and the average of three point measurements.

30 Days in SBF	Ca (%at.)		P (%at.)		O (%at.)		Ca/P	
Place of Analysis	Image Area	Point	Image Area	Point	Image Area	Point	Image Area	Point
NiTi (mechanically ground surface)	12.89	20.88 ± 0.48	12.16	18.52 ± 0.59	38.41	37.92 ± 1.33	1.06	1.13
NiTi + TiO_2_ (290 °C, mechanically ground surface)	12.63	17.15 ± 0.32	11.01	17.97 ± 0.24	44.38	43.38 ± 1.07	1.15	0.95
NiTi + TiO_2_ (190 °C, mechanically ground surface)	13.03	33.63 ± 0.42	11.65	18.94 ± 0.27	42.16	27.49 ± 1.47	1.12	1.78
NiTi + TiO_2_ (390 °C, mechanically ground surface)	10.59	35.61 ± 0.41	10.77	20.13 ± 0.26	40.15	23.81 ± 1.32	0.98	1.76

## Data Availability

Some of the data are contained within the article and some are available on request from the corresponding author (the data are not publicly available due to technical and organizational restrictions).
